# Impact of wildfires on soil microbial nutrient functions in Karst forest ecosystems

**DOI:** 10.3389/fmicb.2026.1765292

**Published:** 2026-01-29

**Authors:** Yuhong Fu, Xu Li, Jianfeng Li, Xun Liu, Yanwei Zhang, Yunlin Zhang

**Affiliations:** 1 Key Laboratory of Ecology and Management on Forest Fire in Higher Education Institutions of Guizhou Province, Guiyang, Guizhou, China; 2 Key Laboratory of Development and Utilization of Biological Resources in Colleges and Universities of Guizhou Province, Guizhou Education University, Guiyang, Guizhou, China; 3 State Key Laboratory of Efficient Production of Forest Resources, School of Ecology and Nature Conservation, Beijing Forestry University, Beijing, China

**Keywords:** forest ecosystems, forest fires, functional types, Karst, soil microorganisms, soil nutrients

## Abstract

This study investigates the impact of wildfires on the diversity and types of soil microbial functions within Karst forest ecosystems, and examines their relationship with soil nutrients. In particular, we focus on the *Quercus fabri* broadleaf and *Pinus massoniana* coniferous forests within areas affected by wildfires in Qiannan, located in the Karst area of Guizhou, Southwestern China. Analysis of soil microbial functional types associated with soil nutrients and their effects was performed using microbial amplicon sequencing technology. Significant differences in the functional diversity of soil bacteria and soil fungi associated with relevant soil nutrients were observed between the *Q. fabri* broadleaf and *P. massoniana* coniferous forests in the study area. After fire, the functional diversity of bacteria in both forest types increased significantly, resulting in a convergence in bacterial functional types. Fire enhanced the functional diversity of fungi in the *P. massoniana* forest; however, had no discernible effect on the *Q. fabri* forest. In addition, fire altered the types and abundance of microbial functions associated with soil nutrients, exerting a greater impact on bacterial functional types. The results also revealed that fire enhanced the abundance of TOC- and TP-related microbial functional types in both forest types, while reducing TK-related functional types. TN-related functional types increased in the *Q. fabri* forest but decreased in the *P. massoniana* forest. At the bacterial level, fire increased TOC-, TN-, and TP-related functional types in both forest types; however, reduced TK-related types. In fungal communities, fire increased TP-related functional groups in the *Q. fabri* forest while reducing TOC-, TN-, and TK-related groups. In contrast, in the *P. massoniana* forest, fire increased TOC- and TP-related groups but decreased TN- and TK-related groups. The research findings provide a scientific basis for the restoration and management of the post-fire forest ecosystems in Karst areas.

## 1 Introduction

With the rising scale, severity, and frequency of wildfires worldwide (Dove et al., 2021), increasing attention is being directed toward post-fire vegetation recovery and the preservation of critical ecosystem services provided by these plants. Fire significantly impacts soil properties and functions within terrestrial ecosystems (Shi et al., 2024). It alters soil organic matter and greatly reduces soil microbial diversity (Steindorff et al., 2022), reducing soil microbial biomass (Garcia-Pausas et al., 2022). Following fire, heterotrophic bacteria in the soil are reported to decrease by 24 and 10% at the 0–3 cm and 5–10 cm soil layers, respectively (Chungopast et al., 2023). Soil microorganisms drive 80–90% of soil processes, making them a pivotal component of soil ecosystems and a key determinant of soil fertility and quality (Saccá et al., 2017). Fire-induced alterations in soil microbial communities significantly impact the structure and function of forest soil microbiomes, thus modifying the microbial activities associated with carbon source substrate utilization (Verma et al., 2019; Cheng et al., 2024a,b). Concurrently, the impact of fire on soil microbes largely determines post-fire soil recovery (Barreiro and Díaz-Raviña, 2021). Rapidly growing soil microbes respond more promptly to fire than soil chemical properties and can influence the recovery of native vegetation communities (Freidenreich et al., 2020). Therefore, investigating the effects of fire on soil microbial diversity, as well as the relationship between microbial diversity and soil quality, can aid in understanding the intrinsic capacity of soils to recover either to their equilibrium or a new equilibrium following s fire (Barreiro and Díaz-Raviña, 2021).

Research indicates that significant differences exist in the physicochemical properties of burned sites during the mid-to-late stages of post-fire recovery in cold-temperate larch forests, altering soil microbial functional diversity (Cheng et al., 2024a). The recovery of soil microbial decomposition activity and functional diversity after fire is closely linked to plant development, as plants typically regenerate by utilizing the increased nutrient availability driven by fires (Garcia-Pausas et al., 2022). Therefore, exploring the functional diversity of post-fire soil microorganisms and the relationship between functional diversity and soil nutrients is necessary for understanding post-fire soil recovery and vegetation restoration. However, research in this area remains relatively scarce, particularly in Karst areas. Karst regions are critical in maintaining biodiversity and providing water resources (Hartmann et al., 2014). The defining feature of Karst environments is susceptible weathering bedrock and shallow soil (Jia et al., 2020, Zhan et al., 2021), rock desertification and its associated ecological fragility (Fu et al., 2016; Vilhar et al., 2022), which if damaged, is extremely difficult to restore (Zhang J. et al., 2024). In China, the karst regions account for 15% of terrestrial land (An et al., 2025). The Karst forest ecosystems in southern China feature unique environmental conditions, highly heterogeneous habitats, rich biodiversity, and diverse forest vegetation types. Coniferous forests are the most widespread among these, primarily comprising species such as *Pinus massoniana*, *Cunninghamia lanceolata*, and *Cupressus funebris*. Broadleaf forests primarily include species such as *Quercus fabri* and *Camellia japonica* (Zhang Y. L. et al., 2024). The Karst topography in Guizhou Province, southern China, is exceptionally well-developed and covers a vast area. Research on soil microorganisms in fire-affected Karst areas remains limited and primarily focuses on microbial community diversity (Yu et al., 2020; Li et al., 2024).

Therefore, this study selected representative broadleaf tree species *Quercus fabri* and coniferous tree species *Pinus massoniana* in the Qiannan Karst area to collect soil samples from both burned and unburned sites. We subsequently analyzed the functional characteristics of soil microorganisms in burned and unburned coniferous-broadleaf forests and examined their association with soil nutrients using 16S rRNA and ITS gene amplicon sequencing. This study aims to provide a basis for soil microbial recovery and ecological restoration in the fire-affected areas of Karst forest ecosystems.

## 2 Materials and methods

### 2.1 Research area overview

The study area was situated in Huishui county of Guizhou province, where the annual mean temperature ranges between 14 and 16 °C and the annual precipitation averages was 1213.4 mm, characterizing a subtropical monsoon humid climate. The region exhibits diverse vegetation types, with yellow soil predominating. Between March and April 2023, a severe forest fire occurred in this area, with a total burned area of 231.42 ha, primarily affecting *Q. fabri* and *P. massoniana*.

### 2.2 Sampling procedure

Soil samples were collected from four types of pure forest stands of white *Q. fabri* and *P. massoniana* forests at burned and unburned sites using 10 × 10 m^2^ plots in November 2023. The four forest types were: unburned *Q. fabri* forest (UQ), burned *Q. fabri* forest (FQ), unburned *P. massoniana* forest (UP), and burned *P. massoniana* forest (FP). Each of the four forest types included five replicates, totaling 20 plots. More detailed information of the sample plots are shown in Supplementary Table S1.

Soil surface samples were collected in each plot using an “S”-shaped pattern at a depth of 10 cm. Five soil samples were taken from each plot, pooled into composite samples, and placed into self-sealing bags. They were then transported in a cooler back to the laboratory. Stones, plant roots, and organic debris were removed from the soil samples, and the remaining material was passed through a 2 mm sieve. The sample was divided into two portions. One portion was immediately flash-frozen in liquid nitrogen and stored at −80 °C for microbial DNA extraction, and the other portion was air-dried at room temperature for soil nutrient analysis.

### 2.3 Determination of soil nutrients and soil microorganisms

Soil nutrient parameters comprised total organic carbon (TOC), total nitrogen (TN), total phosphorus (TP), and total potassium (TK). Methods for soil nutrient determination, DNA extraction, amplification sequencing, bioinformatics analysis, and the sequencing results for soil microorganisms, are described in Li et al. (2024). The bacterial and fungal ASVs obtained from sequencing were classified and annotated. Bacterial and fungal ecological functions were predicted using the FAPROTAX database (Louca et al., 2016) and FUNGuild tool (Nguyen et al., 2016), respectively.

### 2.4 Data statistical analysis

Retrieve all predicted functional types for bacteria and fungi through Web of Science^1^ and China National Knowledge Infrastructure (CNKI).^2^ Bacterial and fungal functional types associated with soil nutrients C, N, P, and K were then selected for the subsequent data analysis. SPSS 26.0 (IBM, SPSS Inc.) was used to test the normality and perform analysis of variance on soil microbial functional data from both burned and unburned forest soils. Calculate the Shannon-Wiener index (H) and Simpson index (D) for functional types of soil bacteria and fungi in different forest types, and Non parametric Wilcoxon rank-sum tests were applied to assess. RDA analysis was conducted on the divisity H and D in relation to soil nutrient contents of TOC, TN, TP, and TK, revealing correlations between microbial functional diversity and soil nutrients. To enhance the accuracy of inter-factor correlations and factor correspondences, Pearson correlation analysis was conducted on all functional types and soil nutrient factors. Functional types exhibiting significant correlations (*p* < 0.05) with soil nutrient factors were identified based on correlation coefficients and significance levels, followed by Mantel test analysis. GraphPad Prism 9.0 was used to analyze and plot the microbial functional profiles and functional diversity before and after fire. Canoco 5.0.2 was used to conduct RDA analysis and plot the soil microbial functional traits and soil nutrient factors. The R (R Core Team) packages vegan, corrplot, ggcor, ggplot2, and dplyr were employed to perform Mantel tests and generate plots for microbial functional types significantly correlated with soil nutrient factors.

## 3 Results and analysis

### 3.1 Microbial soil nutrient functional diversity

**Figure 1:**
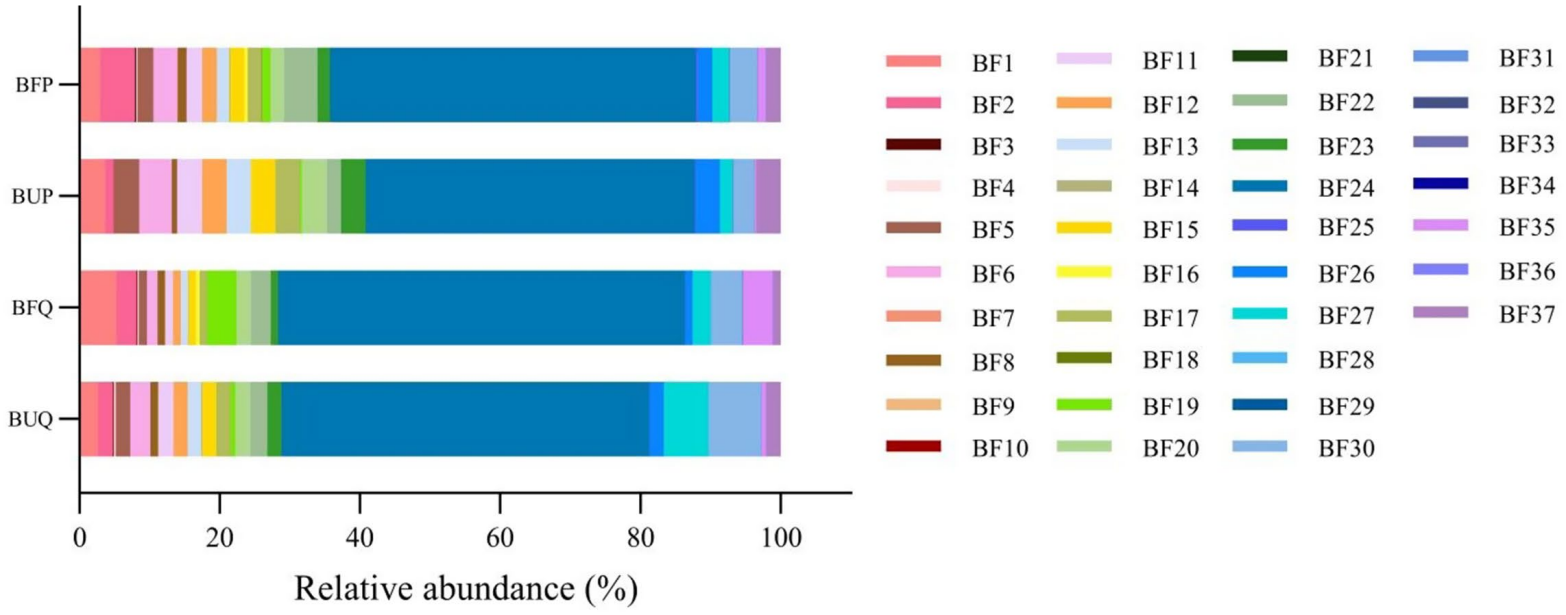
Bacterial functional types associated with soil nutrients in undisturbed and post-fire *Q. fabri* and *P. massoniana* forests. BF1, Respiration of Sulfur Compounds; BF2, Nitrate Ammonification; BF3, Nitrate Reduction; BF4, Nitrate Respiration; BF5, Nitrite Ammonification; BF6, Nitrite Denitrification; BF7, Nitrous Oxide Denitrification; BF8, Nitrogen Respiration; BF9, Nitrogen Fixation; BF10, Nitrate Denitrification; BF11, Nitrite Respiration; BF12, Chemoheterotrophy; BF13, Phototrophy; BF14, Aerofic Chemoheterotrophy; BF15, Chitinolysis; BF16, Aliphatic Non Methane Hydrocarbon Degradation; BF17, Aromatic Hydrocarbon Degradation; BF18, Hydrocarbon Degradation; BF19, Predatory or Exoparasitic; BF20, Sulfate Respiration; BF21, Iron Respiration; BF22, Ureolysis; BF23, Photoheterotrophy; BF24, Xylanolysis; BF25, Methanol Oxidation; BF26, Cellulolysis; BF27, Methanotrophy; BF28, Aromatic Compound Degradation; BF29, Methylotrophy; BF30, Oxygenic Photoautotrophy; BF31, Anoxygenic Photoautotrophy; BF32, Anoxygenic Photoautotrophy S Oxidizing; BF33, Denitrification; BF34, Ligninolysis; BF35, Photoautotrophy; BF36, Cyanobacteria; BF37, Fermentation.

A total of 37 functional types associated with soil nutrients (C, N, P, and K) were identified in both burned and unburned forest soils, the results are shown in Figure 1. The relative abundances of the top 5 of soil bacteria functional types in the unburned *Q. fabri* forest (BUQ) were: xylanolysis, 52.45%; oxygenic photoautotrophy, 7.46%; methanotrophy, 6.40%; nitrite denitrification, 2.85%; and respiration of sulfur compounds, 2.68%. In the burned *Q. fabri* forest, the top 5 of the soil bacteria functional types (BFQ) were: xylanolysis, 57.99%; respiration of sulfur compounds, 5.27%; oxygenic photoautotrophy, 4.40%; predatory or exoparasitic, 4.22%; and photoautotrophy, 4.22%. The relative abundances of the top 5 of soil bacteria functional types in the unburned *P. massoniana* forest (BUP) were: xylanolysis, 46.94%; nitrite denitrification, 4.63%; respiration of sulfur compounds, 3.69%; nitrite ammonification, 3.64%; and nitrite respiration, 3.64%. In the burned *P. massoniana* forest, the top 5 soil bacteria functional types (BFP) were: xylanolysis, 52.19%; nitrate ammonification, 4.96%; ureolysis, 4.76%; oxygenic photoautotrophy, 4.01%; and nitrite denitrification, 3.43%. The primary functional types of soil bacteria in the two forest types differed both before and after fire, and also varied within the same stand. In both *Q. fabri* and *P. massoniana* forests, xylanolysis was a highly abundant functional type in both burned and unburned soils, and its relative abundance increased after fire occurrence. In the *Q. fabri* forest, the relative abundance of respiration of sulfur compounds, predatory or exoparasitic, and photoautotrophy increased after fire, while the relative abundance of oxygenic photoautotrophy, methanotrophy, and nitrite denitrification decreased. In the burned *P. massoniana* forest, the relative abundance of nitrate ammonification, ureolysis, and oxygenic photoautotrophy increased, while the relative abundance of nitrite denitrification, respiration of sulfur compounds, nitrite ammonification, and nitrite respiration decreased.

**Figure 2:**
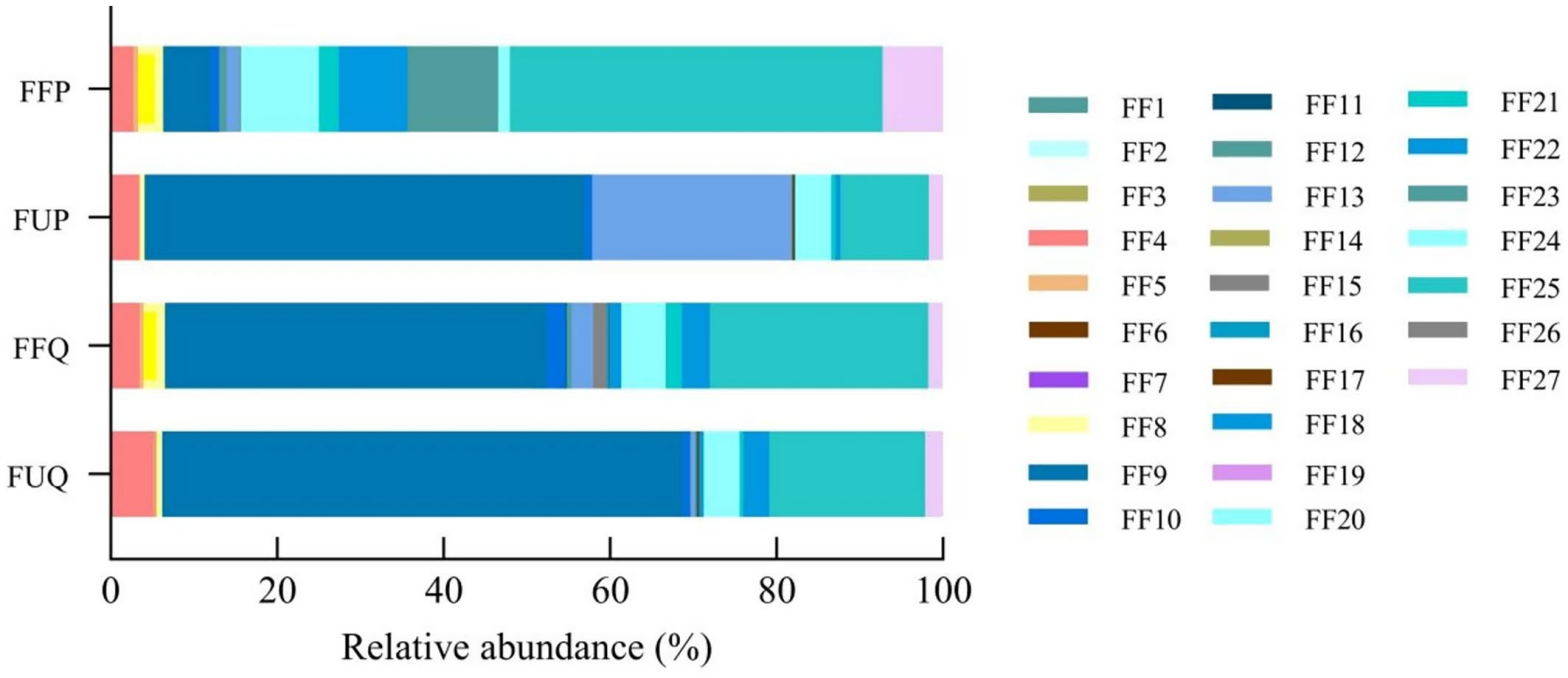
Fungal functional types associated with soil nutrients in undisturbed and post-fire *Q. fabri* and *P. massoniana* forests. FF1, Algal Parasite; FF2, Animal Endosym Fiont; FF3, Animal Parasite; FF4, Animal Pathogen; FF5, ArFuscular Mycorrhizal; FF6, Fryophyte Parasite; FF7, Clavicipitaceous Endophyte; FF8, Dung Saprotroph; FF9, Ectomycorrhizal; FF10, Endophyte; FF11, Epiphyte; FF12, Ericoid Mycorrhizal; FF13, Fungal Parasite; FF14, Leaf Saprotroph; FF15, Lichen Parasite; FF16, Lichenized; FF17, Litter Saprotroph; FF18, Orchid Mycorrhizal; FF19, Plant Parasite; FF20, Plant Pathogen; FF21, Plant Saprotroph; FF22, Soil Saprotroph; FF23, Undefined Fiotroph; FF24, Undefined Parasite; FF25, Undefined Saprotroph; FF26, Undefined Sym Fiotroph; FF27, Wood Saprotroph.

A total of 27 functional types related to soil nutrients were identified in both burned and unburned forest soils, the results are shown in Figure 2. The relative abundances of the top 5 of the soil fungi functional types in the unburned *Q. fabri* forest (FUQ) were: ectomycorrhizal, 62.56%; undefined saprotroph, 18.69%; animal pathogen, 5.10%; plant pathogen, 4.32%; and soil saprotroph, 3.10%. In the burned *Q. fabri* forest, the top 5 of the soil fungi functional types (FFQ) were: ectomycorrhizal, 45.84%; undefined saprotroph, 26.19%; plant pathogen, 5.34%; animal pathogen, 3.38%; and soil saprotroph, 3.29%. The relative abundances of the top 5 of the soil fungi functional types (FUP) in the unburned *P. massoniana* forest were: ectomycorrhizal, 52.91%; fungal parasite, 23.93%; undefined saprotroph, 10.59%; plant pathogen, 4.33%; and animal pathogen, 3.33%. In the burned *P. massoniana* forest, the top 5 of the soil fungi functional types (FFP) were: undefined saprotroph, 44.80%; undefined biotroph, 10.89%; plant pathogen, 9.38%; soil saprotroph, 8.30%; and wood saprotroph, 7.25%. The primary soil fungi functional types in the two forest types differed before and after fire. The primary functional types of soil fungi in burned and unburned *Q. fabri* forests remained unchanged, although their relative abundances varied. Both ectomycorrhizal and undefined saprotroph were highly abundant. After fire, the relative abundance of ectomycorrhizal fungi decreased markedly, while that of undefined saprotroph increased. Simultaneously, the relative abundance of plant pathogen increased, while that of animal pathogen decreased. Comparative analysis reveals pronounced shifts in dominant fungal functional types between burned and unburned soils in the *P. massoniana* forest. In particular, before fire, ectomycorrhizal and fungal parasite were the predominant high-abundance types, while after fire, undefined saprotroph dominated. Following fire, the relative abundances of undefined saprotroph, undefined biotroph, plant pathogen, soil saprotroph, and wood saprotroph increased significantly, while the relative abundances of ectomycorrhizal and fungal parasite exhibited a marked decline. Thus, fires exerted a greater impact on the primary functional types of soil fungi in the *P. massoniana* forest compared to the *Q. fabri* forest.

**Figure 3:**
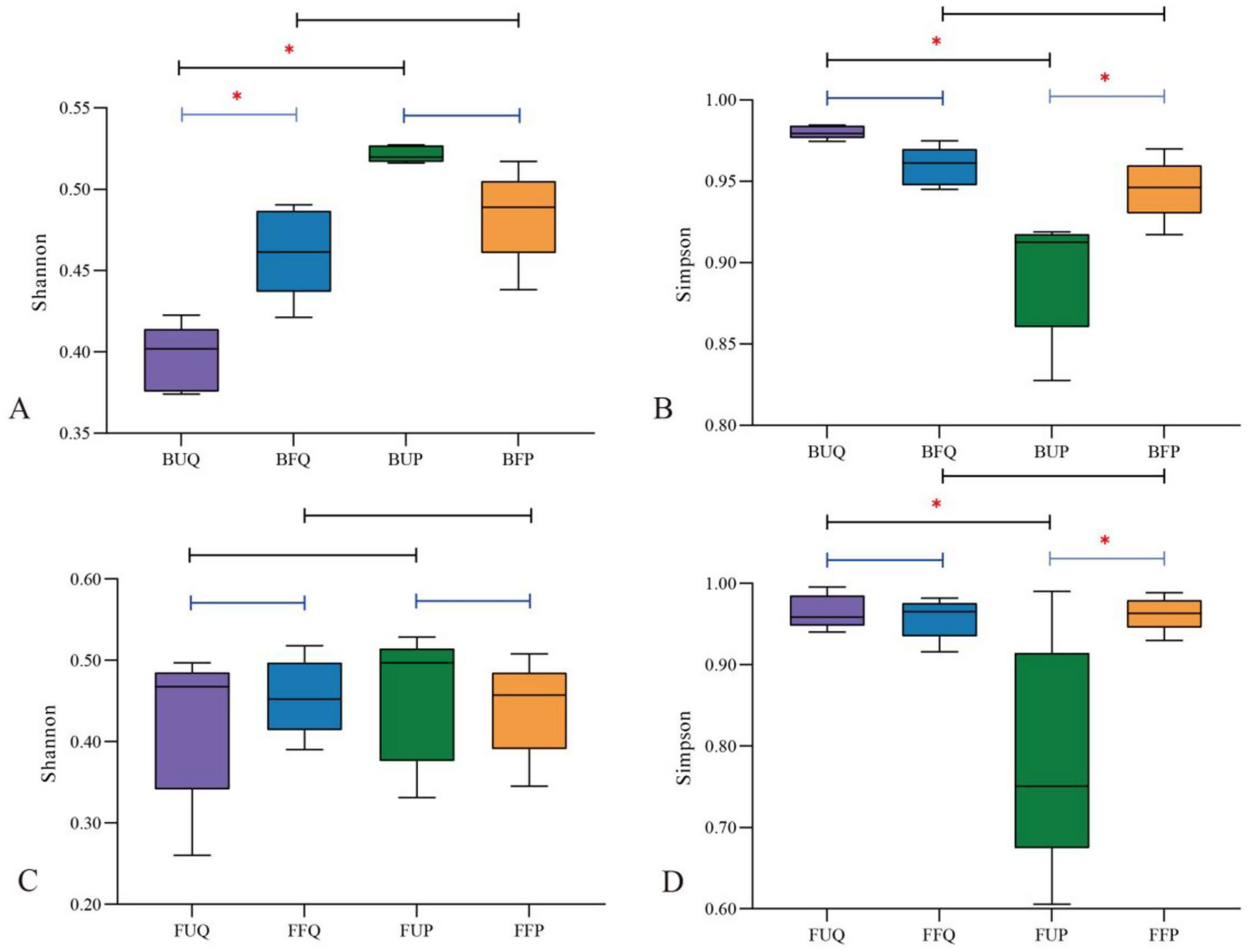
Differences in soil microbial functional diversity between post-fire and undisturbed *Quercus fabri* and *Pinus massoniana* forests (**A,B**: bacteria; **C,D**: fungi. *p < 0.05).

The nonparametric Wilcoxon rank-sum test was performed to analyze differences in soil microbial functional diversity between unburned and burned soils in the two forest stands, the results are shown in Figure 3. In terms of bacterial functional diversity, the Shannon and Simpson indices exhibited significant differences between *Q. fabri* and *P. massoniana* forests before fire. In contrast, no significant differences were observed after fire, this indicates the presence of distinct differences in soil bacterial functional diversity between the two forest types; however, fire significantly alters soil bacterial functional diversity and promotes its convergence. The Shannon index showed significant differences before and after fire in the *Q. fabri* forest, with a marked increase in post-fire conditions. Similarly, the Simpson index exhibited significant differences pre- and post-fire in the *P. massoniana* forest, also showing a pronounced increase after fire, this indicates that fire enhanced bacterial functional diversity in both forest types (Figures 3A,B). In terms of fungal functional diversity, no significant differences were observed in the Shannon index between unburned *Q. fabri* and *P. massoniana* forests, while differences were noted in the Simpson index. After fire, neither index exhibited significant differences, this indicates that soil fungal functional diversity also varied between the two forest types, and that fire alters soil fungal functional diversity. There were no significant differences in the Shannon and Simpson indices pre- and post-fire in the *Q. fabri* forest. In contrast, the Simpson index for the *P. massoniana* forest showed significant differences, with values increasing after fire, this reveals that burning exerted minimal effect on soil fungal functional diversity in the *Q. fabri* forest, while it enhanced diversity in the *P. massoniana* forest (Figures 3C,D).

### 3.2 Relationship between microbial soil nutrient functional diversity and soil nutrients

**Figure 4:**
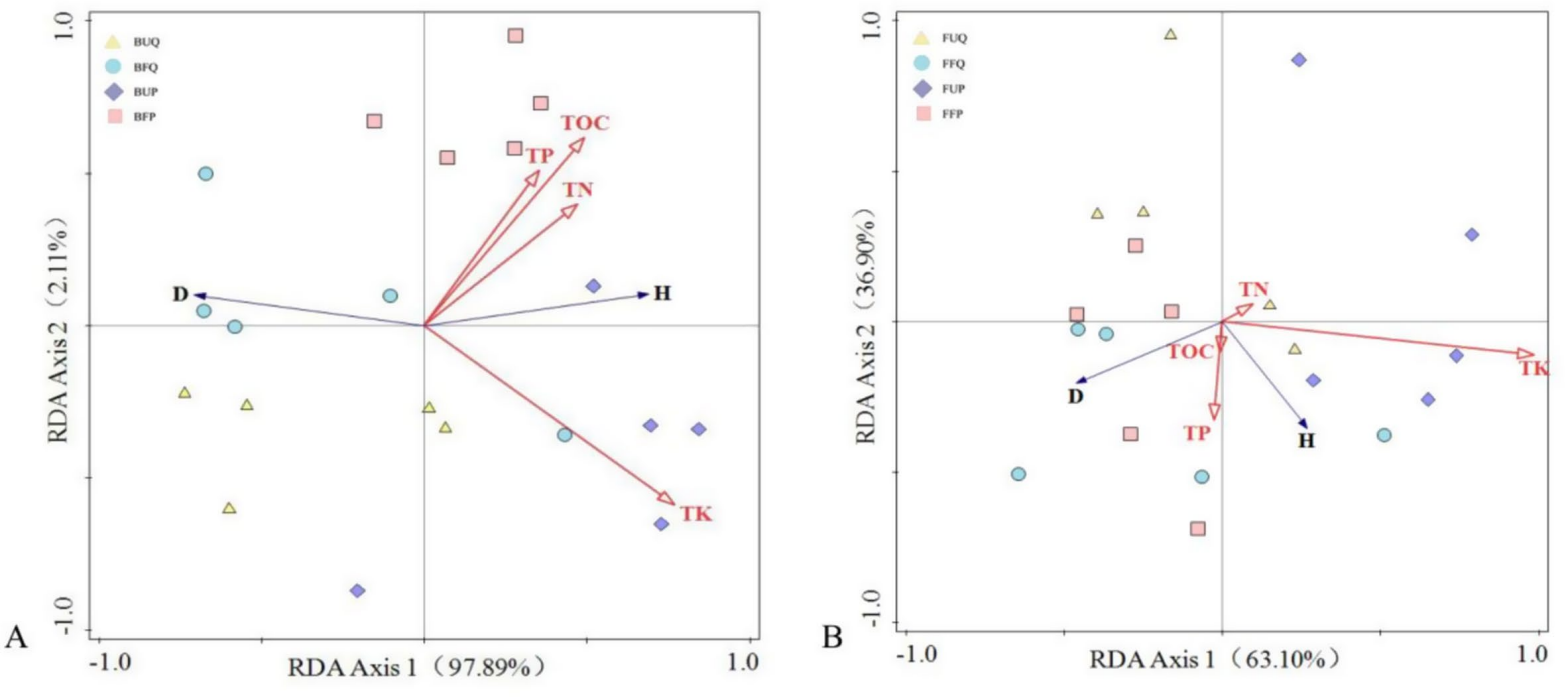
RDA analysis of soil microbial functional diversity and soil nutrient factors (**A**: bacteria; **B**: fungi).

RDA analysis was performed to examine the relationships between soil microbial functional diversity, represented by the Shannon (H) and Simpson (D) indices, and soil nutrient content (TOC, TN, TP, and TK) (Supplementary Table S2). The results indicate that both diversity indices were closely correlated with soil nutrients (Figure 4). Figure 4A presents the relationship between soil bacterial functional diversity and soil nutrients. RDA Axis1 and RDA Axis2 explain 97.89 and 2.11% of the variance, respectively, with both axes accounting for 100% of the variation. H and D exhibited strong positive and negative correlations with four soil nutrient factors, respectively, indicating that soil bacterial functional diversity exerts a significant influence on these four soil nutrient factors. Figure 4B depicts the relationship between soil fungal functional diversity and soil nutrients. RDA Axis1 and RDA Axis2 explain 63.10 and 36.90% of the variance, respectively, with both axes accounting for 100% of the variance. H showed a positive correlation with all four soil nutrient factors. D was positively correlated with TOC and TP, and negatively correlated with TN and TK. This suggests that soil fungal functional diversity also influences these four soil nutrient factors, exhibiting a stronger correlation with TK and relatively weaker correlations with TOC and TN.

### 3.3 Relationship between microbial soil nutrient function type and soil nutrients

**Figure 5:**
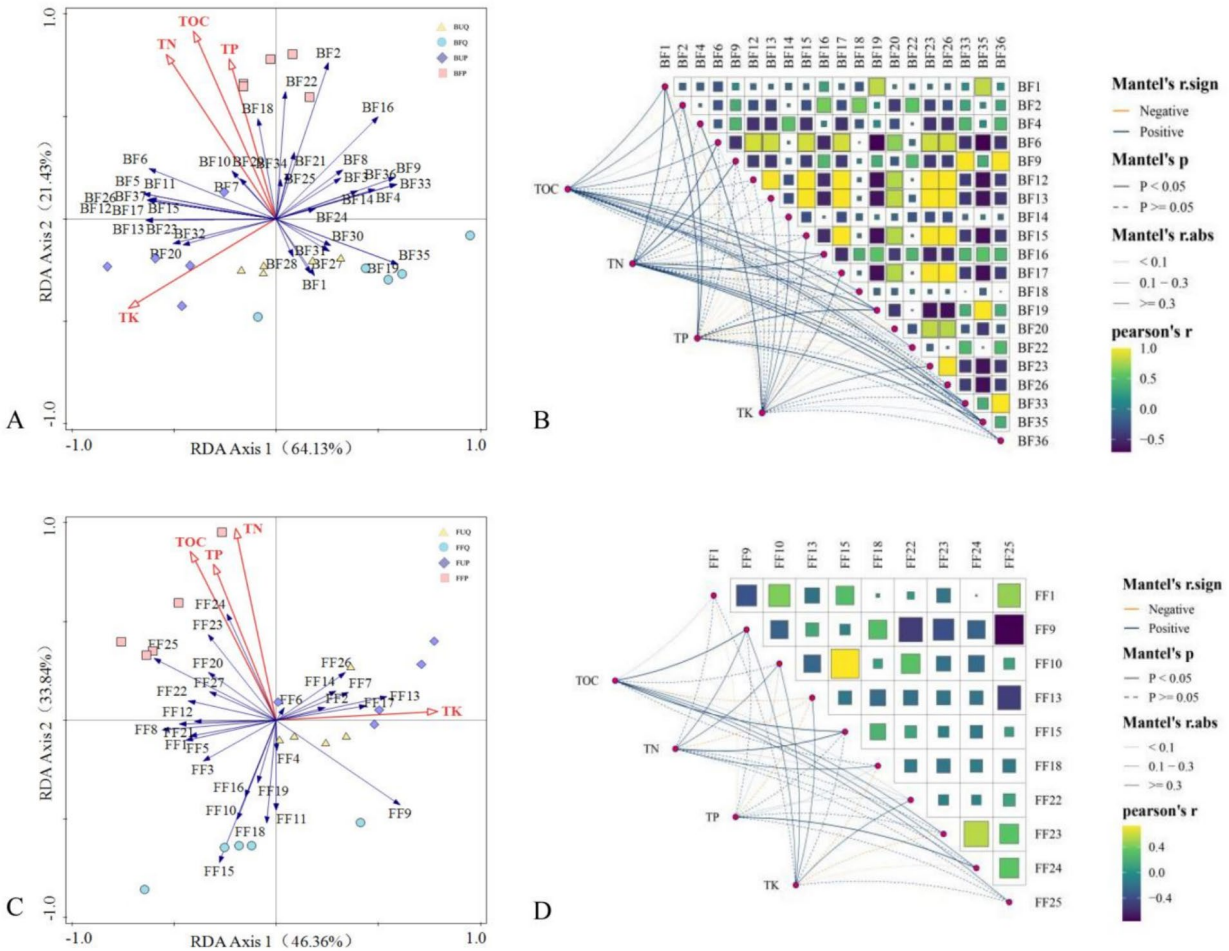
RDA and mantel test analyses between soil microbial functional groups and soil nutrient factors (**A,B**: RDA and mantel test for bacteria; **C,D**: RDA and mantel test for fungi).

RDA analysis and Mantel tests were conducted to examine the relationship between soil microbial functional types and the content of the four soil nutrient factors. Figure 5 presents the results.

In terms of bacterial functional types, RDA analysis revealed that all 37 functional types correlated with the four soil nutrient factors. RDA Axis 1 and RDA Axis 2 explained 64.13 and 21.43% of the variance, respectively, with the two axes accounting for 85.56% of the variation (Figure 5A). To enhance the accuracy of the factor correlations and factor correspondence, Pearson correlation analysis was conducted on all factors (Supplementary Table S3). We selected 20 bacterial functional types exhibiting significant correlations (*p* < 0.05) with the content of the four soil nutrient factors for Mantel test analysis. The Mantel test results indicate that soil bacterial functional types can influence soil nutrient factors, with some functional types simultaneously affecting multiple soil nutrient factors (Figure 5B). Among these bacterial functional types, 6 factors significantly influenced TOC, 5 factors significantly influenced TN, 3 factors significantly influenced TP, 14 factors significantly influenced TK. Nitrate ammonification (BF2) exerted a pronounced effect on TOC, TN, TP, and TK; nitrite denitrification (BF6), predatory or exoparasitic (BF19), and photoautotrophy (BF35) exerted significant effects on TOC and TN; ureolysis (BF22) exerted significant effects on TOC and TP; and respiration of sulfur compounds (BF1) exerted significant effects on TN and TP. In addition, hydrocarbon degradation (BF18) only effect on TOC; nitrate respiration (BF4), nitrogen fixation (BF9), chemoheterotrophy (BF12), phototrophy (BF13), aerobic chemoheterotrophy (BF14), chitinolysi (BF15), aliphatic non methane hydrocarbon degradation (BF16), aromatic hydrocarbon degradation (BF17), sulfate respiration (BF20), photoheterotrophy (BF23), cellulolysis (BF26), denitrification (BF33), and cyanobacteria (BF36) only effect on TK.

In terms of fungal functional types, RDA analysis revealed that all 27 fungal functional types correlated with the four soil nutrient factors. RDA Axis 1 and RDA Axis 2 explained 46.36 and 33.84% of the variance, respectively, with the two axes collectively accounting for 80.20% of the variation (Figure 5C). Person correlation analysis was conducted for all factors (Supplementary Table S3), identifying 10 fungal functional types to correlate significantly (*p* < 0.05) with the four soil nutrient factors for the subsequent Mantel test analysis. The Mantel test results reveal that soil fungal functional types exert similar influences on soil nutrient factors, with certain functional types simultaneously affecting multiple soil nutrient factors (Figure 5D). Among these fungal functional types, 5 factors significantly influenced TOC, 6 factors significantly influenced TN, 2 factors significantly influenced TP, 5 factors significantly influenced TK. Ectomycorrhizal (FF9) exerted a significant influence on TOC, TN, and TK; undefined biotroph (FF23) and undefined parasite significantly affected TOC, TN, and TP; lichen parasite (FF15) significantly impacted TOC and TN; while undefined saprotroph (FF25) significantly affected TOC and TK. In addition, endophyte (FF10) and orchid mycorrhizal (FF18) only effect on TN; algal parasite (FF1), fungal parasite (FF13), and soil saprotroph (FF22) only effect on TK.

### 3.4 Effects of fire on soil nutrient-related microbial functional types

**Figure 6:**
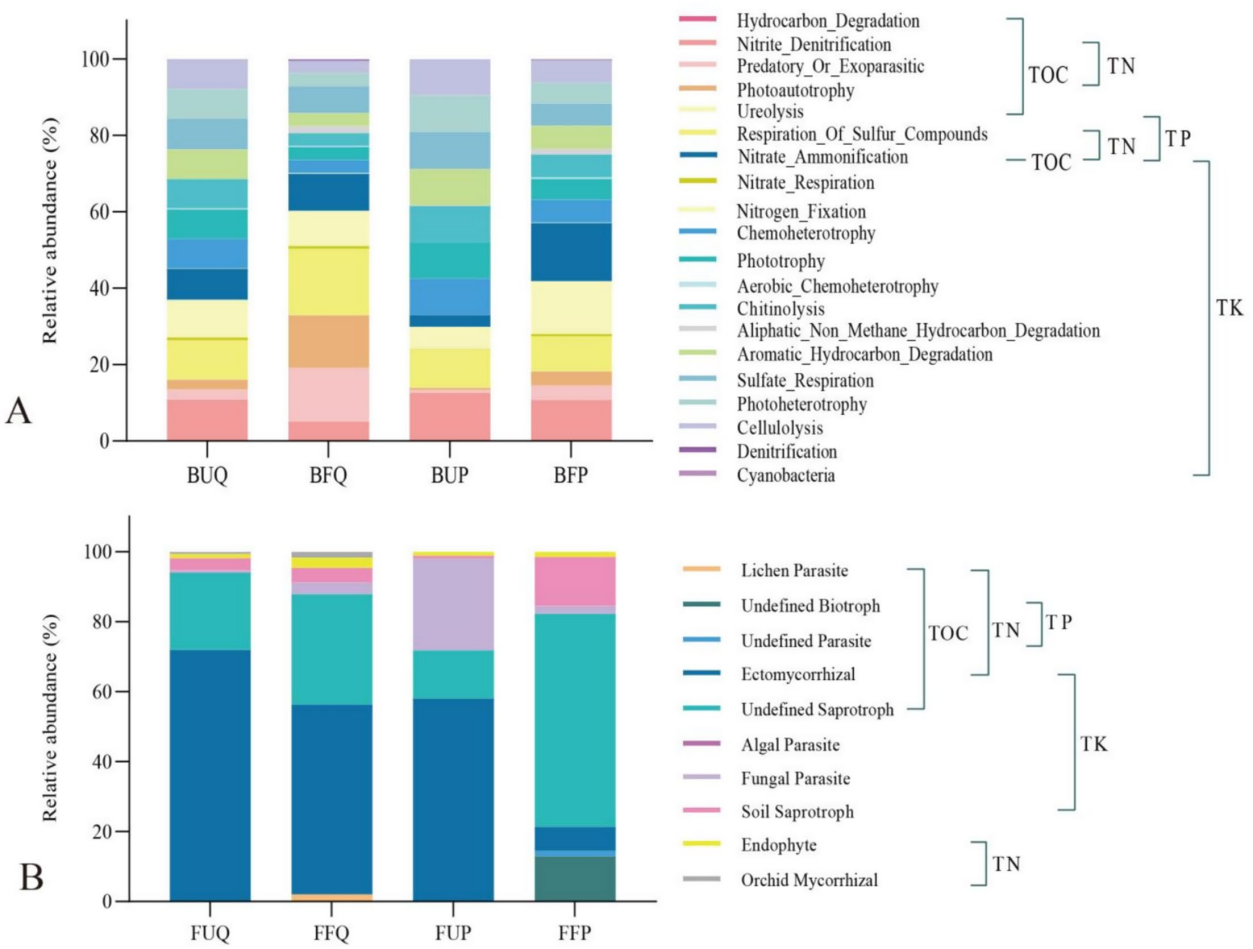
Abundance of soil microbial functional types showing significant correlations to soil nutrient factors across two forest stands before and after firing (**A**: bacteria; **B**: fungi).

The relative abundances of 20 bacterial functional types were significantly correlated with soil nutrient factors in the two forest stands pre- and post-fire (Figure 6A). In terms of relative abundances of the top 5 of soil bacteria functional types in two types of forests before and after fires. Post fires, the relative abundance of respiration of sulfur compounds (relevant TN, TP), predatory or exoparasitic (relevant TOC, TN), photoautotrophy (relevant TOC, TN), and nitrate ammonification (relevant TOC, TN, TP, TK) in *Q. fabri* forest soil increased, while the relative abundance of nitrite denitrification (relevant TOC, TN), ureolysis (relevant TOC, TP), and sulfate respiration (relevant TK) decreased. After fire, the relative abundance of nitrate ammonification (relevant TOC, TN, TP, TK) and ureolysis (relevant TOC, TP) in *P. massoniana* forest soil increased substantially, while nitrite denitrification (relevant TOC, TN), respiration of sulfur compounds (relevant TN, TP), sulfate respiration (relevant TK), chemoheterotrophy (relevant TK), and chitinolysis (relevant TK) decreased. Soil bacterial functional types varied between unburned *Q. fabri* and *P. massoniana* forests; however, nitrite denitrification and respiration of sulfur compounds remained the most abundant functional types. Post fire, the high-abundance bacteria functional types shifted in both forest types. In particular, predatory or exoparasitic and photoautotrophy became dominant in the *Q. fabri* forest, while nitrate ammonification and ureolysis emerged as dominant in the *P. massoniana* forest. In terms of soil bacterial functional group relative abundance, the unburned *Q. fabri* forest exhibited the following TOC-related relative abundance totals: 34.09% for TOC, 34.52% for TN, 28.22% for TP, and 63.85% for TK. The corresponding post-fire values were 51.90% for TOC, 60.01% for TN, 36.21% for TP, and 40.52% for TK (Supplementary Table S4). This indicates that after fire, the relative abundance of bacterial functional types associated with soil TOC, TN, and TP increased, while TK relative abundance decreased. In the unburned *P. massoniana* forest, the combined relative abundances associated with TOC, TN, TP, and TK were 22.87, 27.14, 19.03, and 70.14%, respectively. The corresponding post-fire values were 47.29, 42.50, 38.08, and 58.88%, respectively (Supplementary Table S4). This indicates that following fire, bacterial functional group relative abundances associated with soil TOC, TN, and TP increased, while those linked to TK decreased.

The relative abundances of 10 fungal functional types were significantly correlated with soil nutrient factors in the two forest types before and after burning (Figure 6B). In terms of relative abundances of the top 5 of soil fungal functional types in two types of forests before and after fires. Following fire, the levels of high-abundance fungal functional types in the *Q. fabri* forest exhibited significant changes. In particular, the relative abundances of undefined saprotroph (relevant TOC, TK), soil saprotroph (relevant TK), fungal parasite (relevant TK), and endophyte (relevant TN) increased, while those of ectomycorrhizal (relevant TOC, TN, TK) and orchid mycorrhizal (relevant TN) decreased. After fire, the levels of high-abundance fungal functional types in the *P. massoniana* forest also changed significantly. Specifically, the relative abundances of undefined saprotroph (relevant TOC, TK), soil saprotroph (relevant TK), and undefined biotroph (relevant TOC, TN, TP) increased greatly, while the relative abundances of ectomycorrhizal (relevant TOC, TN, TK) and fungal parasite (relevant TK) declined substantially. In terms of soil fungal functional types, differences were observed between unburned *Q. fabri* and *P. massoniana* forests. However, neither exhibited ectomycorrhizal nor undefined saprotroph as dominant functional types, with ectomycorrhizal being absolutely predominant (more than 58%). The key distinction was the presence of a highly abundant dominant functional type—fungal parasite—in the *P. massoniana* forest. Following fire, the two forest types exhibited minimal shifts in high-abundance fungal functional types but significant variations in relative abundance. In the *Q. fabri* forest, ectomycorrhizal and undefined saprotroph remained dominant, with fungal parasite emerging as the predominant type, while undefined biotroph became the dominant functional type within the *P. massoniana* forest. In terms of the functional type relative abundances of soil fungi, the unburned *Q. fabri* forest exhibited total TOC, TN, TP, and TK relative abundances of 94.03, 73.80, 0.03, and 98.05%, respectively. Post fire, the corresponding relative abundances were 87.85, 60.90, 0.12, and 93.23%, respectively (Supplementary Table S5). This indicates that after fire, fungal functional group relative abundances associated with soil TOC, TN, and TK decreased, while those associated with TP increased. In the unburned *P. massoniana* forest, the total relative abundances associated with TOC, TN, TP, and TK were 71.81, 59.25, 0.02, and 98.80%, respectively. Following fire, the corresponding relative abundances were 82.26, 22.98, 14.41, and 83.93%, respectively (Supplementary Table S5). This reveals that post-fire, the relative abundances of fungal functional types associated with soil TOC and TP increased, whereas those associated with TN and TK decreased.

## 4 Discussion

### 4.1 Effects of fire on soil nutrient-related microbial functional diversity

Studies indicate that fire significantly impacts the structure and function of forest soil microorganisms (Dove et al., 2021; Garcia-Pausas et al., 2022; Cheng et al., 2024a; Li et al., 2024). However, the impact of fires on the functional diversity and functional types of soil microorganisms across different forest stands remains unclear. Our results reveal that the functional diversity of soil bacteria and fungi differed between unburned *Q. fabri* broadleaf and *P. massoniana* coniferous forests, with more pronounced differences in bacterial functional diversity. Fire significantly increased soil bacterial functional diversity in both forest types and promoted convergence in bacterial functional types, although xylanolysis remained the dominant functional type before and after fire. Moreover, fire exerted a greater influence on the primary fungal functional types and enhanced its diversity in the *P. massoniana* coniferous forest, while no significant effects were observed in the *Q. fabri* broadleaf forest. Both forest types exhibit a greater numerous in bacterial functional types compared to fungal functional types. These results are attributed to the strong and varying impacts of fire on the soil nutrient content across both forest types. Typically, fires reduce soil organic carbon, nitrogen, phosphorus, and potassium (Verma et al., 2019). However, they also rapidly decompose surface litter and accelerate nutrient influx into the soil, thereby increasing nutrient content (Cheng et al., 2024b). This study determined post-fire values of TOC, TN, and TP contents to decrease in the soil of the *Q. fabri* broadleaf forest, while no significant changes were observed in the TK content. Conversely, the TOC, TN, and TP contents in the *P. massoniana* coniferous forest soil increased, while the TK content decreased (Supplementary Table S2). Thus, post-fire soil nutrient changes differed between *Q. fabri* broadleaf and *P. massoniana* coniferous forests. This can be attributed to variations in litter type and accumulation. In the *P. massoniana* forest, the predominance of slow-decomposing coniferous litter leads to greater accumulation of surface litter buildup. Following fire, this results in a larger nutrient influx into the soil, consequently raising the TOC, TN, and TP levels. Previous research indicates that changes in post-fire soil microbial functional diversity are closely related to soil nutrient content, including soil organic carbon and available nitrogen (Li et al., 2021). Accordingly, differences in post-fire soil nutrient content between the two forest types led to variations in soil microbial functional diversity.

### 4.2 Effects of fire on nutrient-related soil microbial functional types

RDA analysis indicates that in both forest types, soil microbial functional diversity and functional types were closely correlated with the four soil nutrient factors. Fire influences soil microbial functional diversity and significantly alters microbial functional types associated with soil nutrients. Our analysis of the soil microbial functional groups significantly correlated with the four soil nutrient factors revealed that burning exerted distinct effects on the high-abundance (top five) bacterial and fungal functional groups in the two forest stands. In particular, fire had a greater impact on bacterial functional types, altering two functional types, while its effect on fungal functional types was smaller, changing only a single functional type. Moreover, the bacterial and fungal functional types affected by fire differed between the two forest stands. In terms of bacterial functional group relative abundance, the total relative abundance in both forest types prior to burning followed the order: TK > TN > TOC > TP. Post fire, the order in the *Q. fabri* forest changed to TN > TOC > TK > TP, while that in the *P. massoniana* forest was TK > TOC > TN > TP. Furthermore, post-burning, the relative abundances of the TOC, TN, and TP functional groups increased in both forest types, while the relative abundances of the TK functional group decreased. The functional type relative abundances in the *Q. fabri* and *P. massoniana* forests were of the order TN > TOC > TP and TOC > TP > TN, respectively. The decline in TK relative abundance was more pronounced in the *Q. fabri* forest compared to the *P. massoniana* forest. The overall fungal functional relative abundance in both forest types before and after fire was of the order: TK > TOC > TN > TP. Post fire, the relative abundances of the TN and TK functional types decreased, while TP relative abundance increased. TOC relative abundance declined in the *Q. fabri* forest and increased in the *P. massoniana* forest. In terms of the total microbial functional group relative abundance (sum of bacterial and fungal functional group relative abundances), both forest types exhibited the order TK > TOC > TN > TP prior to burning. After fire, the *P. massoniana* forest maintained the same order, whereas that of the *Q. fabri* forest shifted to TOC > TK > TN > TP, although TOC and TK remained closely matched. Post fire, both forest types exhibited increased TOC and TP relative abundances and reduced TK relative abundance. TN relative abundance increased in the *Q. fabri* forest and decreased in the *P. massoniana* forest. These results indicate that prior to fire, both forest types exhibited the highest activity in TK functional types and the lowest in TP. Fire subsequently enhanced the abundances of TOC and TP functional types in both forest types, while reducing the TK functional type abundance. The TN functional type increased in the *Q. fabri* forest but decreased in the *P. massoniana* forest; however, following fire, the TK functional type remained highly active, while the TP functional type remained comparatively low. Fire exerted distinct effects on the bacterial and fungal functional types within the two forest stands, with a more pronounced impact on bacterial functional types. Research indicates that in terms of soil nutrient cycling driven by soil microorganisms, fires significantly alter the carbon source utilization capacity of soil microbes (Cheng et al., 2024a). Our research confirms that burning enhances soil carbon nutrient cycling in both forest types, although fungal carbon functional types decline in the *Q. fabri* forest. This enhancement in carbon nutrient cycling primarily relies on bacterial functional types. Fire significantly heightened phosphorus nutrient cycling in both forest types. Although both bacterial and fungal functional types exhibited greater activity, the improvement in phosphorus cycling was primarily attributed to bacterial functional types. In contrast, fire reduced soil potassium nutrient cycling in both forest types, research indicates potassium is a primary factor limiting microbial proliferation (Ganjegunte et al., 2005). Therefore, reduced soil potassium nutrient cycling can promotes the recovery of soil microorganisms after burning. For soil nitrogen cycling, fire led to a decline in the *P. massoniana* forest but an increase in the *Q. fabri* forest, with bacterial functional types increasing in both forest types while fungal functional types decreased, while bacterial functional types in the *Q. fabri* forest increased relatively more and fungal functional types decreased relatively less. Note that in both forest stands, potassium exhibited the highest relative nutrient cycling, while phosphorus showed the lowest, both before and after the fire. It is evident that following fire, the microbial-driven soil nutrient restructuring process in both forest types primarily enhances carbon and phosphorus nutrients, particularly phosphorus and nitrogen nutrient dynamics. However, variations occur between forest types, potentially linked to differing fire impacts on bacterial and fungal functional types, as well as distinct post-fire soil nitrogen contents across the two forest communities. In addition, the trends in soil bacterial functional type relative abundance for post-fire C, N, P, and K were consistent across both forest stands, aligning with the convergence of soil bacterial functional diversity following burning. A comprehensive analysis of post-fire changes in soil bacterial and fungal functional types and their abundances associated with C, N, and P indicates that bacterial functional types play a pioneering role in soil nutrient restructuring following fire. This may be attributed to the greater sensitivity of bacteria compared with fungi to changes in the soil environment, enabling the rapid utilization of newly available resources for reproduction and metabolism post-fire (Wang and Kuzyakov, 2024).

In our research, the microbial amplicon sequencing technology was used to predict soil microbial functions, which is a common method in soil microbial research. Although this method has the limitation that it only predicts possible potential functions of microorganisms indirectly, it is still helpful for us to understand the basic functional overview or trends of soil microorganisms on the whole. Meanwhile, our research only explored the correlations between four main soil nutrients and soil microbial functional types on two types of foersts before and after fire. Moreover, due to the lack of continuous sampling and measurement, our research was unable to reflect dynamic changes of soil microbial functional types related to four types of soil nutrients under the influence of forest fires. In addition, factors such as geography, climate, slope position, and altitude all can influence or regulate the diversity of soil microorganisms and their distribution patterns (Yang et al., 2023; Chen et al., 2025; Guo et al., 2025). Therefore, in order to reveal comprehensively the response mechanisms of soil nutrient-related microbial functional types to forest fires, it is necessary to do further study on temporal and spatial changes of soil microbial functional types after fires, and the correlations between more soil nutrients and influencing factors and soil microbial functional types in future research.

## 5 Conclusion

The functional diversity of soil bacteria and fungi associated with relevant soil nutrients in *Q. fabri* broadleaf and *P. massoniana* coniferous forests varied significantly before fire. Fire significantly increased bacterial functional diversity in both forest types and promoted the convergence in bacterial functional types. Moreover, fire enhanced fungal functional diversity in the *P. massoniana* forest; however, had no apparent effect on the *Q. fabri* forest. Soil microbial functional diversity and functional types in both forest types were closely correlated with soil C, N, P, and K nutrients. We observed fire to alter the types and abundance of microbial functions associated with soil nutrients, exerting a greater impact on bacterial functional types. Fire increased the abundance of TOC, TN, and TP bacterial functional types in both forest types while reducing TK abundance. It also increased the abundance of TP-related fungal functional types in the *Q. fabri* forest while reducing TOC-, TN-, and TK-related abundances. In the *P. massoniana* forest, fire increased TOC- and TP-related fungal functional types, while decreasing TN- and TK-related abundances. In terms of total microbial functional group abundance, fire increased the overall abundance of TOC- and TP-related functional types in both forest types, while reducing TK-related functional types. TN-related functional types increased in the *Q. fabri* forest but decreased in the *P. massoniana* forest. These findings provide valuable insights into understanding microbial-driven soil nutrient recovery following forest fires in Karst areas and offer a scientific basis for post-fire forest ecosystem restoration and management.

## Data Availability

The original contributions presented in the study are included in the article/Supplementary material, further inquiries can be directed to the corresponding authors.

## Footnotes

1
http://www.webofknowledge.com

2
https://www.cnki.net
