# Supplementary material for: Impact of wildfires on soil microbial nutrient functions in Karst forest ecosystems
Source: Front Microbiol. 2026 Jan 29;17:1765292. doi: 10.3389/fmicb.2026.1765292 (PMC12894227; doi:10.3389/fmicb.2026.1765292)
Supplement: Supplementary file 1 [file Table_1.docx]

**Appendix Table 1**

**General description of the control and burned sites**

| **Site** | UQ | FQ | UP | FP |
| --- | --- | --- | --- | --- |
| **Burned year** | Unburned | 2023 | Unburned | 2023 |
| **Type** | *Quercus fabri* | *Quercus fabri* | *Pinus massoniana* | *Pinus massoniana* |
| **Vegetation type** | Arbor (*Quercus fabri*) | Herbage (*Imperata cylindrica*) | Arbor (*Pinus massoniana*) | Herbage (*Pteridium aquilinum*) |
| **Latitude(N)** | 26°2′44′′ | 26°2′51′′ | 26°4′57′′ | 26°4′54′′ |
| **Longitude(E)** | 106°53′13′′ | 106°53′10′′ | 106°39′48′′ | 106°39′56′′ |
| **Elevation (m)** | 1179 | 1170 | 1089 | 1131 |
| **Aspect** | Sunny | Sunny | Sunny | Sunny |

Notes: UQ: Unburned *Quercus fabri* forest; FQ: Burned *Quercus fabri* forest.

UP: Unburned *Pinus massoniana* forest; FP: Burned *Pinus massoniana* forest.

**Appendix Table 2**

**Differences in soil nutrient content between two forest stands Before and After Fire**

| **Name** | **TOC（g/kg）** | **TN（g/kg）** | **TP（g/kg）** | **TK（g/kg）** |
| --- | --- | --- | --- | --- |
| **FQ** | 68.175±7.215b | 1.346±0.214b | 0.204±0.047b | 2.615±1.429a |
| **UQ** | 114.41±12.599a | 2.69±0.301a | 0.336±0.046a | 2.648±0.931a |
| **FP** | 290.199±17.743a | 4.807±0.589a | 0.674±0.29a | 1.949±0.535b |
| **UP** | 166.154±31.784b | 3.31±0.339b | 0.365±0.058b | 4.581±0.877a |

*p*<0.05

**Appendix Table 4**

**Abundance of soil bacterial functional types showing significant correlations to soil nutrient factors across two forest stands before and after fire**

| **BUQ** | **ID** | **Functional Types** | **Relative Abundance（%）** |
| --- | --- | --- | --- |
|  | BF6 | Nitrite Denitrification | 10.974 |
|  | BF1 | Respiration of Sulfur Compounds | 10.242 |
|  | BF22 | Ureolysis | 9.804 |
|  | BF2 | Nitrate Ammonification | 8.176 |
|  | BF20 | Sulfate Respiration | 8.144 |
|  | BF12 | Chemoheterotrophy | 7.677 |
|  | BF13 | Phototrophy | 7.677 |
|  | BF15 | Chitinolysis | 7.677 |
|  | BF17 | Aromatic Hydrocarbon Degradation | 7.677 |
|  | BF23 | Photoheterotrophy | 7.677 |
|  | UBF26 | Cellulolysis | 7.677 |
|  | BF19 | Predatory or Exoparasitic | 2.566 |
|  | BF35 | Photoautotrophy | 2.566 |
|  | BF4 | Nitrate Respiration | 0.824 |
|  | BF14 | Aerobic Chemoheterotrophy | 0.302 |
|  | BF9 | Nitrogen Fixation | 0.087 |
|  | BF33 | Denitrification | 0.087 |
|  | BF36 | Cyanobacteria | 0.087 |
|  | BF16 | Aliphatic Non Methane Hydrocarbon Degradation | 0.081 |
|  | BF18 | Hydrocarbon Degradation | 0.000 |

| **BFQ** | **ID** | **Functional Types** | **Relative Abundance（%）** |
| --- | --- | --- | --- |
|  | BF1 | Respiration of Sulfur Compounds | 17.300 |
|  | BF19 | Predatory or Exoparasitic | 13.912 |
|  | BF35 | Photoautotrophy | 13.912 |
|  | BF2 | Nitrate Ammonification | 9.719 |
|  | BF22 | Ureolysis | 9.192 |
|  | BF20 | Sulfate Respiration | 6.998 |
|  | BF6 | Nitrite Denitrification | 5.164 |
|  | BF12 | Chemoheterotrophy | 3.388 |
|  | BF13 | Phototrophy | 3.388 |
|  | BF15 | Chitinolysis | 3.388 |
|  | BF17 | Aromatic Hydrocarbon Degradation | 3.388 |
|  | BF23 | Photoheterotrophy | 3.388 |
|  | BF26 | Cellulolysis | 3.388 |
|  | BF16 | Aliphatic Non Methane Hydrocarbon Degradation | 1.821 |
|  | BF4 | Nitrate Respiration | 0.823 |
|  | BF14 | Aerobic Chemoheterotrophy | 0.293 |
|  | BF9 | Nitrogen Fixation | 0.180 |
|  | BF33 | Denitrification | 0.180 |
|  | BF36 | Cyanobacteria | 0.180 |
|  | BF18 | Hydrocarbon Degradation | 0.000 |

| **BUP** | **ID** | **Functional Types** | **Relative Abundance（%）** |
| --- | --- | --- | --- |
|  | BF6 | Nitrite Denitrification | 12.721 |
|  | BF1 | Respiration of Sulfur Compounds | 10.099 |
|  | BF20 | Sulfate Respiration | 9.787 |
|  | BF12 | Chemoheterotrophy | 9.513 |
|  | BF15 | Chitinolysis | 9.513 |
|  | BF17 | Aromatic Hydrocarbon Degradation | 9.513 |
|  | BF26 | Cellulolysis | 9.513 |
|  | BF13 | Phototrophy | 9.491 |
|  | BF23 | Photoheterotrophy | 9.491 |
|  | BF22 | Ureolysis | 5.825 |
|  | BF2 | Nitrate Ammonification | 3.109 |
|  | BF19 | Predatory or Exoparasitic | 0.608 |
|  | BF35 | Photoautotrophy | 0.608 |
|  | BF16 | Aliphatic Non Methane Hydrocarbon Degradation | 0.106 |
|  | BF4 | Nitrate Respiration | 0.102 |
|  | BF18 | Hydrocarbon Degradation | 0.000 |
|  | BF9 | Nitrogen Fixation | 0.000 |
|  | BF14 | Aerobic Chemoheterotrophy | 0.000 |
|  | BF33 | Denitrification | 0.000 |
|  | BF36 | Cyanobacteria | 0.000 |

| **BFP** | **ID** | **Functional Types** | **Relative Abundance（%）** |
| --- | --- | --- | --- |
|  | BF2 | Nitrate Ammonification | 15.244 |
|  | BF22 | Ureolysis | 13.755 |
|  | BF6 | Nitrite Denitrification | 10.731 |
|  | BF1 | Respiration of Sulfur Compounds | 9.076 |
|  | BF12 | Chemoheterotrophy | 6.049 |
|  | BF15 | Chitinolysis | 6.049 |
|  | BF17 | Aromatic Hydrocarbon Degradation | 6.049 |
|  | BF26 | Cellulolysis | 6.049 |
|  | BF20 | Sulfate Respiration | 5.887 |
|  | BF13 | Phototrophy | 5.350 |
|  | BF23 | Photoheterotrophy | 5.350 |
|  | BF19 | Predatory or Exoparasitic | 3.726 |
|  | BF35 | Photoautotrophy | 3.726 |
|  | BF16 | Aliphatic Non Methane Hydrocarbon Degradation | 1.429 |
|  | BF4 | Nitrate Respiration | 0.716 |
|  | BF14 | Aerobic Chemoheterotrophy | 0.406 |
|  | BF18 | Hydrocarbon Degradation | 0.107 |
|  | BF9 | Nitrogen Fixation | 0.106 |
|  | BF33 | Denitrification | 0.098 |
|  | BF36 | Cyanobacteria | 0.098 |

**Appendix Table 5**

**Abundance of soil fungal functional types showing significant correlations to soil nutrient factors across two forest stands before and after fire**

| **FUQ** | **ID** | **Functional Types** | **Relative Abundance（%）** |
| --- | --- | --- | --- |
|  | FF9 | Ectomycorrhizal | 71.853 |
|  | FF25 | Undefined Saprotroph | 22.116 |
|  | FF22 | Soil Saprotroph | 3.482 |
|  | FF10 | Endophyte | 1.191 |
|  | FF18 | Orchid Mycorrhizal | 0.692 |
|  | FF13 | Fungal Parasite | 0.580 |
|  | FF15 | Lichen Parasite | 0.032 |
|  | FF1 | Algal Parasite | 0.020 |
|  | FF23 | Undefined Biotroph | 0.019 |
|  | FF24 | Undefined Parasite | 0.015 |

| **FFQ** | **ID** | **Functional Types** | **Relative Abundance（%）** |
| --- | --- | --- | --- |
|  | FF9 | Ectomycorrhizal | 54.127 |
|  | FF25 | Undefined Saprotroph | 31.529 |
|  | FF22 | Soil Saprotroph | 4.280 |
|  | FF13 | Fungal Parasite | 3.271 |
|  | FF10 | Endophyte | 2.883 |
|  | FF15 | Lichen Parasite | 2.079 |
|  | FF18 | Orchid Mycorrhizal | 1.694 |
|  | FF23 | Undefined Biotroph | 0.064 |
|  | FF24 | Undefined Parasite | 0.053 |
|  | FF1 | Algal Parasite | 0.020 |

| **FUP** | **ID** | **Functional Types** | **Relative Abundance（%）** |
| --- | --- | --- | --- |
|  | FF9 | Ectomycorrhizal | 58.058 |
|  | FF13 | Fungal Parasite | 26.200 |
|  | FF25 | Undefined Saprotroph | 13.728 |
|  | FF10 | Endophyte | 1.141 |
|  | FF22 | Soil Saprotroph | 0.817 |
|  | FF18 | Orchid Mycorrhizal | 0.034 |
|  | FF23 | Undefined Biotroph | 0.011 |
|  | FF15 | Lichen Parasite | 0.006 |
|  | FF24 | Undefined Parasite | 0.005 |
|  | FF1 | Algal Parasite | 0.000 |

| **FFP** | **ID** | **Functional Types** | **Relative Abundance（%）** |
| --- | --- | --- | --- |
|  | FF25 | Undefined Saprotroph | 60.816 |
|  | FF22 | Soil Saprotroph | 14.009 |
|  | FF23 | Undefined Biotroph | 12.821 |
|  | FF9 | Ectomycorrhizal | 6.906 |
|  | FF13 | Fungal Parasite | 2.169 |
|  | FF24 | Undefined Parasite | 1.587 |
|  | FF10 | Endophyte | 1.510 |
|  | FF15 | Lichen Parasite | 0.128 |
|  | FF18 | Orchid Mycorrhizal | 0.027 |
|  | FF1 | Algal Parasite | 0.027 |
